# Supplementary material for: Architecture of the U6 snRNP reveals specific recognition of 3′-end processed U6 snRNA
Source: Nat Commun. 2018 May 1;9:1749. doi: 10.1038/s41467-018-04145-4 (PMC5931518; doi:10.1038/s41467-018-04145-4)
Supplement: Supplementary file 1 — Supplementary Information [file 41467_2018_4145_MOESM1_ESM.pdf]

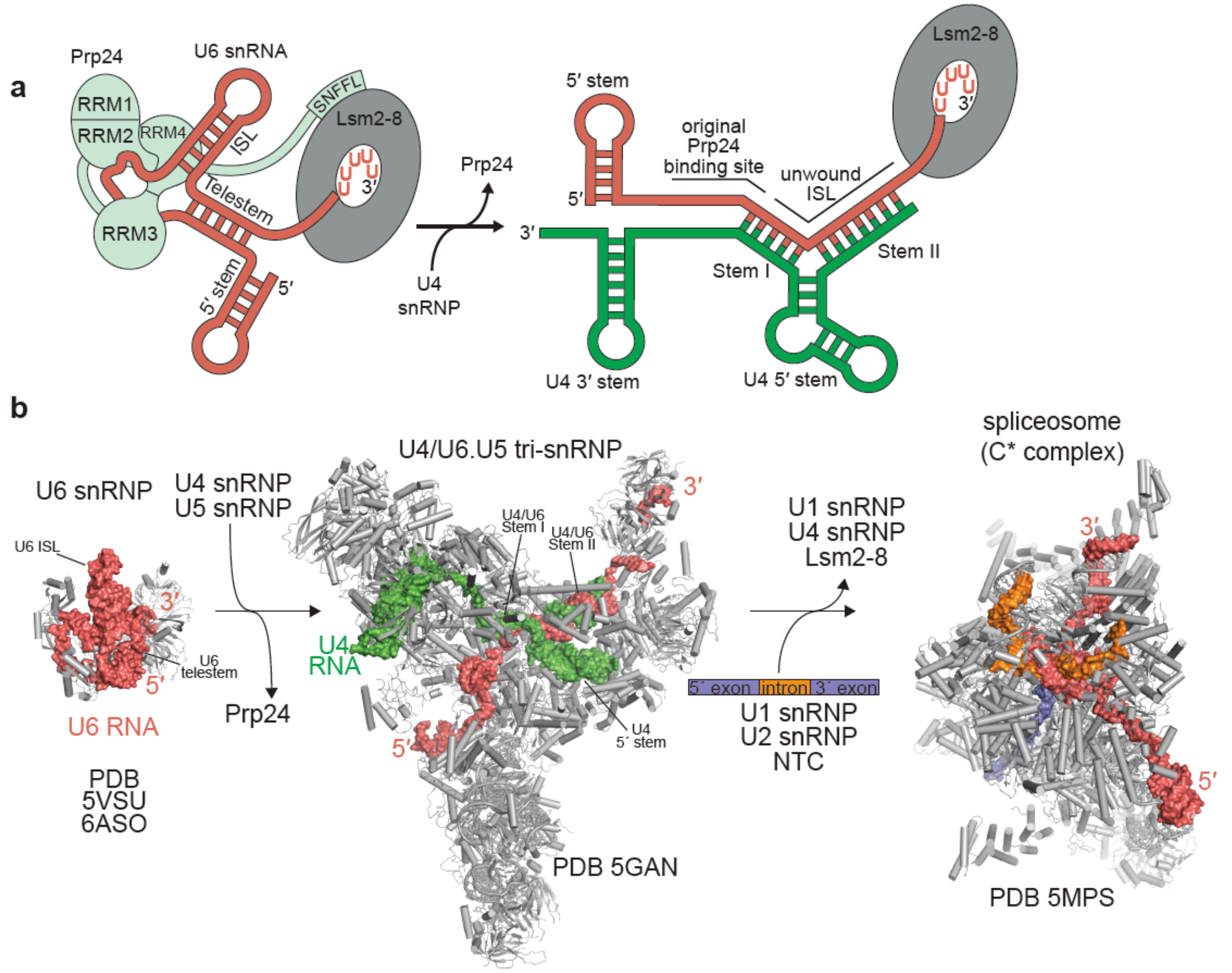

Supplementary Fig 1 Overview of U6 snRNA structure and assembly into the spliceosome. **a** Simplified cartoon representation of the yeast U6 and U4/U6 snRNPs. For clarity, U4-associated proteins are not depicted. Prp24 binds U6 and catalyzes formation of the U4/U6 snRNP, which requires unwinding of the U6 internal stem loop (ISL). Lsm2-8 is known to bind the conserved C-terminal “SNFFL box” motif in Prp24 and the 3′ oligouridylate tract in U6, and accelerates Prp24-mediated annealing of U4/U6 through an unknown mechanism[1-3]. **b** U6 snRNA undergoes extensive structural remodeling during pre-mRNA splicing. U6 is highly compact in the U6 snRNP due to extensive intramolecular base-pairing. In contrast, U6 is highly extended in the U4/U6.U5 tri-snRNP, in which Prp24 is absent but the Lsm2-8 ring is still bound to U6. In activated spliceosomes the Lsm2-8 ring is gone, the ISL is mostly reformed, and U6 makes numerous protein-RNA contacts, in addition to pairing with U2 snRNA.

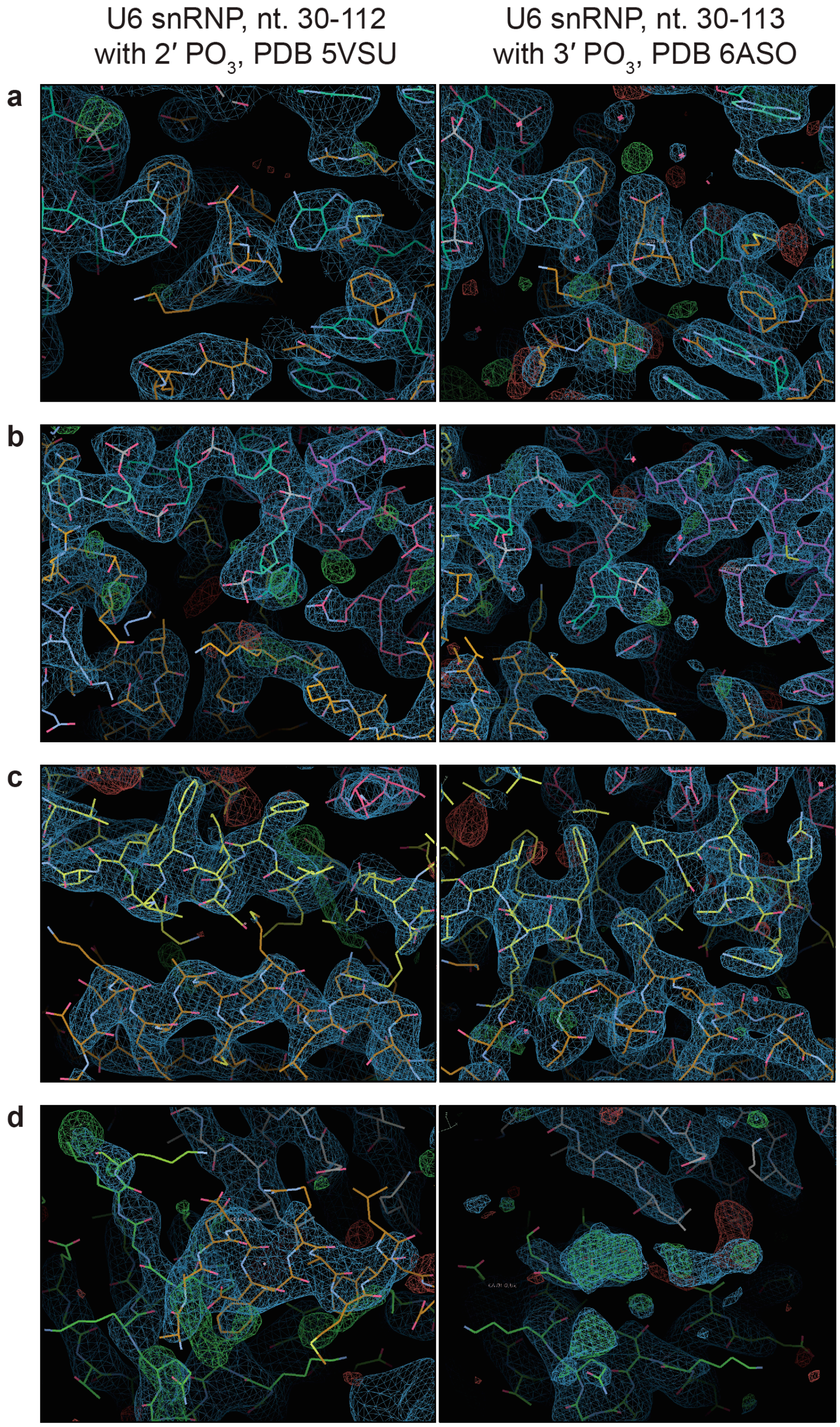

Supplementary Fig 2 Representative electron density. Blue density is of form 2mF_o-DF_c and is contoured at 1.5 rmsd. Positive and negative density (colored green and red, respectively) is of form mF_o-DF_c and is contoured at 3.0 rmsd. **a** Density for the core of the U6-Prp24 interaction, centered at Asp288 (the aspartate bridge[4]) and U6 nucleotides A42 and G55. **b** Density for the 3′ tail of U6 RNA bound inside the Lsm2-8 ring. **c** Density for the Prp24-RRM4/Lsm2 interface. **d** Density for the Prp24-SNFFL box interaction with Lsm5 and Lsm7. Alpha helical density was modeled as the SNFFL box in PDB 5VSU in space group $P2_12_12_1$. Due to weak density, the SNFFL box was not modeled into PDB 6ASO in space group $P2_1$.

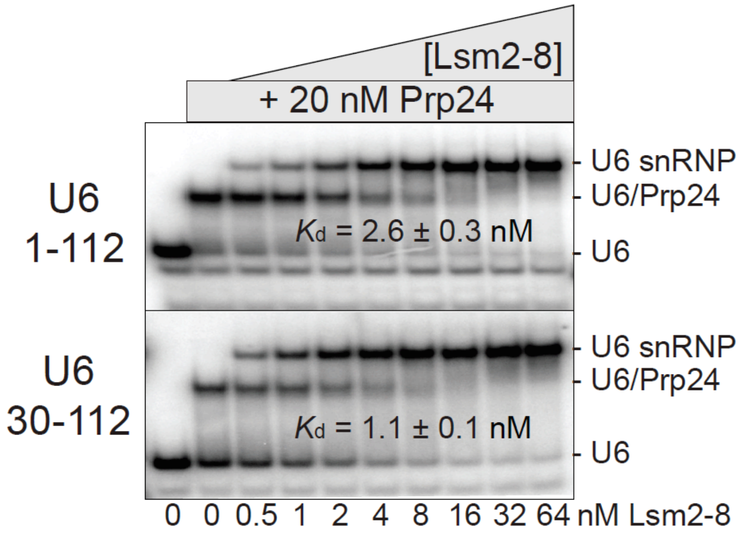

Supplementary Fig 3 The 5′ stem of U6 does not contribute to U6 snRNP stability *in vitro*. Electrophoretic mobility shift was used to monitor formation of U6 snRNPs in the presence of 20 nM Prp24 and varying concentrations of Lsm2-8, using RNA that had been 5′ labeled with ^{32}P. K_d is the dissociation constant for Lsm2-8 from the ternary complex (Prp24•U6•Lsm2-8). Note that the binary complex of Prp24•U6 appears to be less stable in the absence of the U6 5′ stem.

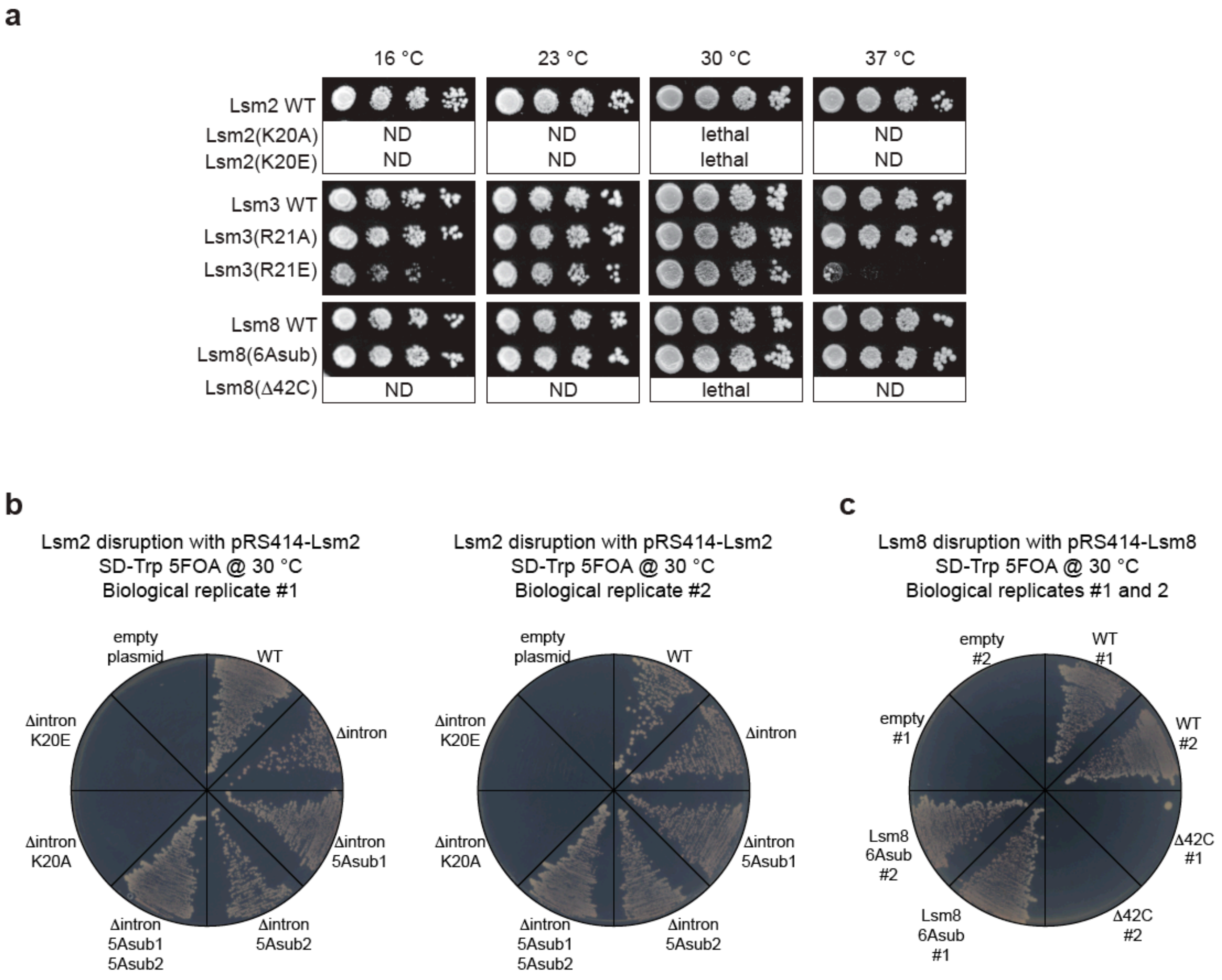

Supplementary Fig 4 Mutation of 3′ end binding residues in Lsm2-8. **a** Point mutant strains exhibit diverse growth phenotypes *in vivo*, which agree well with *in vitro* binding defects (Table 3). ND = not tested due to lethality during initial 5-FOA selection at 30 °C. **b** The intron in Lsm2 is not essential to viability, and mutation of Lsm2 residue K20 is lethal. The 5Asub1 and 5Asub2 alleles contain Lsm2 substitutions at the Prp24/Lsm2 interface (5Asub1 = S5A/K8A/T9A/V11A/D12A, 5Asub2 = L81A/D84A/R88A/E89A/T92A). **c** Removal of the C-terminal 42 residues of Lsm8 is also lethal. The Lsm8 6Asub allele contains K87A, D88A, T89A, K90A, N91A and K92A substitutions at the Lsm8 contact with the 3′ end of U6 snRNA. Lethality was determined via 5-FOA counter-selection of the wild-type Lsm alleles on solid media lacking tryptophan at 30 °C. Two independent transformants were tested for each allele.

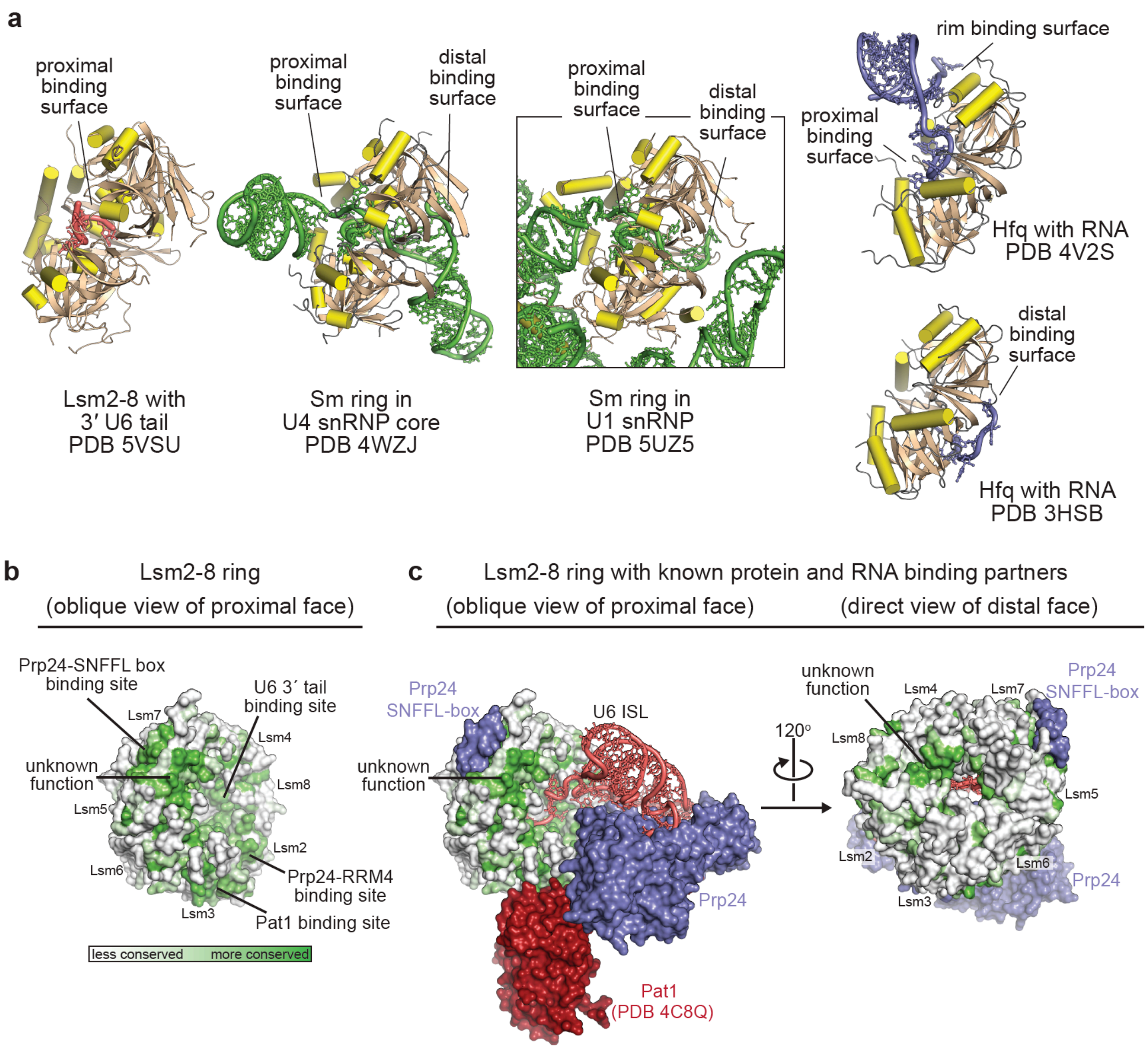

Supplementary Fig 5 Homology and surface conservation in protein-protein and protein-RNA binding surfaces of the Lsm2-8 ring. **a** Comparison of known binding mechanisms between RNA and Lsm2-8 or homologous protein ring structures. Unlike the Lsm2-8 ring[5], the U1 and U4 snRNAs are threaded through the homologous Sm ring[6,7] in the U1 and U4 snRNPs, respectively, and employ a mixture of proximal, rim, and distal RNA binding mechanisms like in the Hfq homologs of the Lsm2-8 ring[8-11]. **b** There are numerous patches of sequence conservation on the proximal and rim surfaces of the Lsm2-8 ring that can be attributed to binding of U6 snRNA and Prp24 in the U6 snRNP, or Pat1 in the Lsm1-7/Pat1 complex[12]. **c** Overlay of known binding partners in the U6 snRNP or Lsm1-7/Pat1 complex shows an additional patch of sequence conservation in the N-terminal region of Lsm5 (approximately residues 7-16 and 37) that is proximal to the bound C-terminal SNFFL-box motif of Prp24. There is an additional patch of sequence conservation along the distal face of the ring between Lsm4 (approximately residues 18-24) and Lsm8 (approximately residues 17-21) that does not interact with Prp24 or U6 in the U6 snRNP, or Pat1 in the Lsm1-7/Pat1 complex, that could provide a binding surface for protein and/or RNA during U4/U6 annealing, or partially comprise the nuclear localization sequence in the Lsm2-8 ring[13]. Conservation was determined with the program ConSurf[14] and the alignment in Supplementary Note 1.

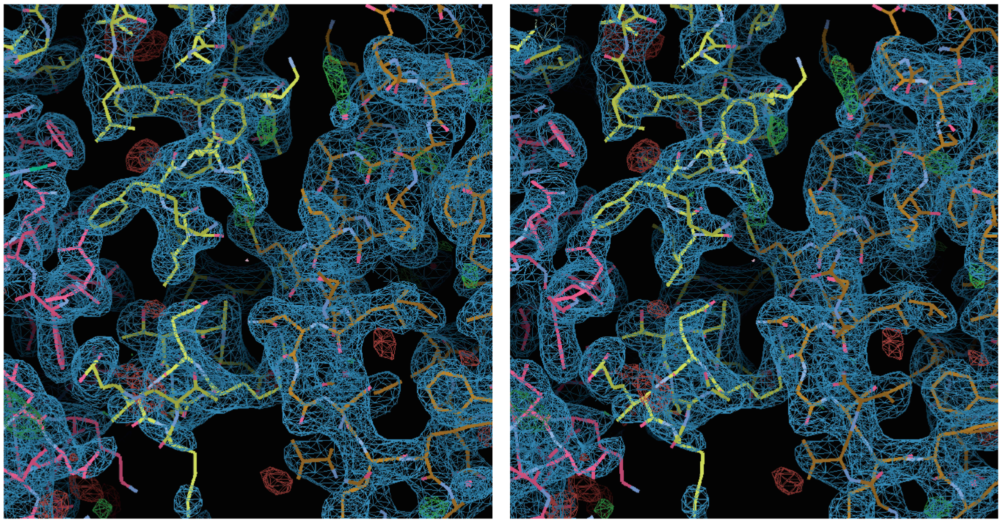

Supplementary Fig 6 Cross-eyed stereo view of electron density at the Lsm2/RRM4 interface. Blue density is of form $2mF_o$-DF_c and is contoured at 1 r.m.s.d. Positive and negative density (colored green and red, respectively) is of form mF_o-DF_c and is contoured at 3.0 r.m.s.d. Prp24 RRM4 is colored orange, Lsm2 is colored yellow, and Lsm3 is colored pink.

Figure 3d

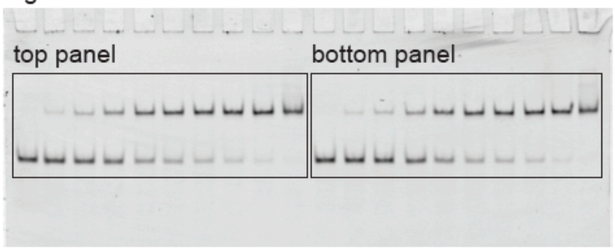

Figure 3e

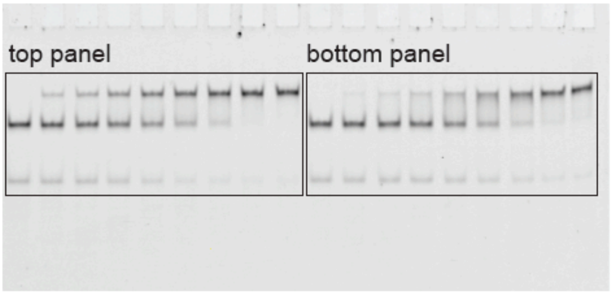

Figure 3f

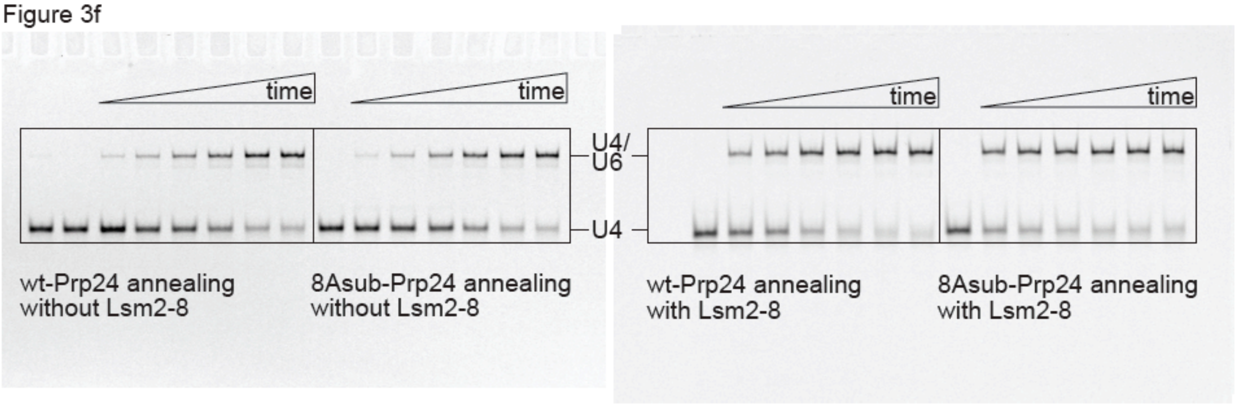

Supplementary Figure 3

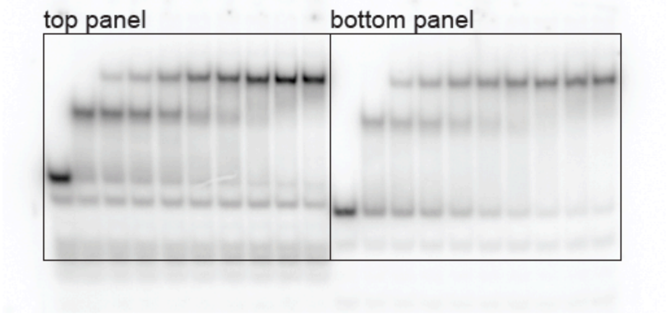

Supplementary Fig 7 Representative, uncropped gel images.

Supplementary Note 1: Sequence alignment of Lsm2-8 proteins

red = truncations used for crystallization, or substitutions tested in this study
grey = disordered in yeast U6 snRNP crystal structure

Lsm2

```
              1        10        20        30        40        50        60        70        80        90        100
              |         |         |         |         |         |         |         |         |         |         |
H.sapiens     MLFYSFFKSLVGKDVVVELKNDLSICGTLHSVDQYLNIKLTDISVTDPEKYPHMLSVKNCFIRGSVVRYVQLPADEVDTQLLQDAARKEALQQKQ-----
B.taurus      MLFYSFFKSLVGKDVVVELKNDLSICGTLHSVDQYLNIKLTDISVTDPEKYPHMLSVKNCFIRGSVVRYVQLPADEVDTQLLQDAARKEALQQKQ-----
S.scrofa      MLFYSFFKSLVGKDVVVELKNDLSICGTLHSVDQYLNIKLTDISVTDPEKYPHMLSVKNCFIRGSVVRYVQLPADEVDTQLLQDAARKEALQQKQ-----
F.catus       MLFYSFFKSLVGKDVVVELKNDLSICGTLHSVDQYLNIKLTDISVTDPEKYPHMLSVKNCFIRGSVVRYVQLPADEVDTQLLQDAARKEALQQKQ-----
C.familiaris  MLFYSFFKSLVGKDVVVELKNDLSICGTLHSVDQYLNIKLTDISVTDPEKYPHMLSVKNCFIRGSVVHYVQLPADEVDTQLLQDAARKEALQQKQ-----
D.rerio       MLFYSFFKSLVGKDVVVELKNDLSICGTLHSVDQYLNIKLTDISVTDPEKYPHMLSVKNCFIRGSVVRYVQLPADEVDTQLLQDAARKEATQQKQ-----
X.laevis      MLFYSFFKSLVGKDVVVELKNDLSICGTLHSVDQYLNIKLTDISVTDPEKYPHMLSVKNCFIRGSVVRYVQLPADEVDTQLLQDAARKEAVQQKQ-----
A.thaliana    MLFFSYFKDLVGQEVTVELKNDLAIRGTLHSVDQYLNIKLENTRVVDQDKYPHMLSVRNCFIRGSVVRYVQLPKDGVDVDLLHDAARREARGG-------
O.sativa      MLFFSYFKELVGKEVTVELKNDLAIRGTLHSVDQYLNIKLENTRVVDQDKYPHMLSVRNCFIRGSVVRYVLLPQDGVDIDILHDATRREARGG-------
S.pombe       MLFYSFFKTLIDTEVTVELKNDMSIRGILKSVDQFLNVKLENISVVDASKYPHMAAVKDLFIRGSVVRYVHMSSAYVDTILLADACRRDLANNKRQ----
C.neoformans  MLIFSFFKTLTDQVITVELKNDLSITGTLKSVDQFLNIRLDNISVEDPERHPHMMAVKNCFIRGSVVRYVRMAARSVDTTLLEDATRREAKEGKK-----
N.crassa      MLFFSFFKTLIDHEVTVELKNDIQIRGVLKSVDQFLNIKLDNIQVVEELKYPHLSAVKNVFIRGSVVRYVHLPQESVDIQLLEDATRREAANQATKAKQG
C.glabrata    MLFFSFFKTLVDQEVVVQLKNDIEIKGTLQSVDQFLNLKLDNISCNDDKKYPHLSSVRNIFIRGSTVRYVYLNKNMVDTNLLQDATRREHLSDRK-----
S.cerevisiae  MLFFSFFKTLVDQEVVVELKNDIEIKGTLQSVDQFLNLKLDNISCTDEKKYPHLGSVRNIFIRGSTVRYVYLNKNMVDTNLLQDATRREVMTERK-----
              **::*:** *  .   :.*:****: * * *:****:**::* :     :  ::**: :*:: *****.*:** :     **   :* ** *::
```

Lsm3

```
              1        10        20        30        40        50        60        70        80        90        100
              |         |         |         |         |         |         |         |         |         |         |
H.sapiens     MADDVDQQQTTNTVEEPLDLIRLSLDERIYVKMRNDRELRGRLHAYDQHLNMILGDVEETVTTIEIDEETYEEIYKSTKRNIPMLFVRGDGVVLVAPPLRVG------
B.taurus      MADDVDQQQTTNTVEEPLDLIRLSLDERIYVKMRNDRELRGRLHAYDQHLNMILGDVEETVTTIEIDEETYEEIYKSTKRNIPMLFVRGDGVVLVAPPLRVG------
S.scrofa      MADDVDQQQTTNTVEEPLDLIRLSLDERIYVKMRNDRELRGRLHAYDQHLNMILGDVEETVTTIEIDEETYEEIYKSTKRNIPMLFVRGDGVVLVAPPLRVG------
F.catus       MADDVDQQQTTNTVEEPLDLIRLSLDERIYVKMRNDRELRGRLHAYDQHLNMILGDVEETVTTIEIDEETYEEIYKSTKRNIPMLFVRGDGVVLVAPPLRVG------
C.familiaris  MADDVDQQQTTNTVEEPLDLIRLSLDERIYVKMRNDRELRGRLHAYDQHLNMILGDVEETVTTIEIDEETYEEIYKSTKRNIPMLFVRGDGVVLVAPPLRVG------
G.gallus      MADEVDQQQTTNTVEEPLDLIRLSLDERIYVKMRNDRELRGRLHAYDQHLNMILGDVEETVTTIEIDEETYEEIYKSTKRNIPMLFVRGDGVVLVAPPLRVG------
D.rerio       MADDAEQQQTTNTVEEPLDLIRLSLDERIYVKMRNDRELRGRLHAYDQHLNMILGDVEETVTTVEIDEETYEEIYKSTKRNIPMLFVRGDGVVLVAPPLRVG------
X.laevis      MADDGEQQQTTNTVEEPLDLIRLSLDERIYVKMRNDRELRGRLNAYDQHLNMILGDVEETVTTIEIDEETYEEIYKSTKRNIPMLFVRGDGVVLVAPPLRVG------
A.thaliana    -----MSGEEEATVREPLDLIRLSLDERIYVKLRSDRELRGKLHAFDQHLNMILGDVEETITTVEIDDETYEEIVRTTKRTIEFLFVRGDGVILVSPPLRTAA-----
O.sativa      (8aa)-AAEEEIAVKEPLDLIRLSLDERIYVKLRSDRELRGKLHAYDQHLNMILGDVEEIVTTVEIDDETYEEIVRTTKRTIPFLFVRGDGVILVSPPLRTA------
S.pombe       -------MESAQAVAEPLDLVRLSLDEIVYVKLRGDRELNGRLHAYDEHLNMVLGDAEEIVTIFDDEETDKDKALKTIRKHYEMLFVRGDSVILIAPPRN--------
C.neoformans  -----MDAVVNSQIQEPLDLVKLALGERVLIKLRGDRIVTGVLHAYDAHMNVVISQAEESIHVVDVTEEGQPLPPRIERRTAEMLFVRGDGVILLSPAEQ--------
N.crassa      ---MADAVEDAGSVSEPMDLVRLLLDEVVCVKLRGDRELKGRLHAYDSHCNLVLGEVEETIYVVDDEDTEE-DDLKTISRKSEMLFVRGDSVVLISPFTRS-------
C.glabrata    -----------MSLSTPLDLLKLNLDERVYVKLRGARAMEGVLQAFDSHCNIVLSDAVETIYELVDG------ELKSQERASEMIFVRGDSVTLITAVSEEE------
S.cerevisiae  -------------METPLDLLKLNLDERVYIKLRGARTLVGTLQAFDSHCNIVLSDAVETIYQLNNE------ELSESERRCEMVFIRGDTVTLISTPSEDDDGAVEI
                           :  *:**::* *.* : :*:*. * : * *:*:* * *:::.:. * :  .              :  ::*:*** * *::   .
```

Lsm4

```
              1        10        20        30        40        50        60        70        80        90        100       110       120       130
              |        |         |         |         |         |         |         |         |         |         |         |         |         |
H.sapiens     MLPLSLLKTAQNHPMLVELKNGETYNGHLVSCDNWMNINLREVICTSRDGDKFWRMPECYIRGSTIKYLRIPDEIIDMVKEEVVAKGRGRGGLQQQKQQKGRGMGGAGRGVFGGRGRGGIPGTGRGQPEKKPGRQAGKQ
B.taurus      MLPLSLLKTAQNHPMLVELKNGETYNGHLVSCDNWMNINLREVICTSRDGDKFWRMPECYIRGSTIKYLRIPDEIIDMVKEEVVAKGRGRGGLQQQKQQKGRGMGGAGRGVFGGRGRGGIPGTGRGQPEKKPGRQAGKQ
F.catus       MLPLSLLKTAQNHPMLVELKNGETYNGHLVSCDNWMNINLREVICTSRDGDKFWRMPECYIRGSTIKYLRIPDEIIDMVKEEVVAKGRGRGGLQQQKQQKGRGMGGAGRGVFGGRGRGGIPGTGRGQPEKKPGRQAGKQ
C.familiaris  MLPLSLLKTAQNHPMLVELKNGETYNGHLVSCDNWMNINLREVICTSRDGDKFWRMPECYIRGSTIKYLRIPDEIIDMVKEEVVAKGRGRGGLQQQKQQKGRGMGGAGRGVFGGRGRGGIPGTGRGQPEKKPGRQAGKQ
G.gallus      MLPLSLLKTAQNHPMLVELKNGETYNGHLVSCDNWMNINLREVICTSRDGDKFWRMPECYIRGSTIKYLRIPDEIIDMVKEEVVSKGRGRGGMQQQKQQKGRGVGGAGRGVFGGRGR-GIPGSGRGQQEKKPGRQSAKQ
D.rerio       MLPLSLLKTAQNHPMLVELKNGETYNGHLVSCDNWMNINLREVICTSRDGDKFWRMPECYIRGSTIKYLRIPDEIIDMVKEEVVSKGRGRGGMQQNKPQ-(14aa)-GRGVFGGRGRGMQGSGRGQQQDKKPGKQQV--
X.laevis      MLPLSLLKTAQNHPMLVELKNGETYNGHLVSCDNWMNINLREVICTSRDGDKFWRMPECYIRGSTIKYLRIPDEIIDMVKEEVMSKGRGRGGMQQQKQMKGRGAGGGGRGVFGGRGR-GAPGGGRGQQDKKPGRQSGKQ
A.thaliana    MLPLSLLKTAQGHPMLVELKNGETYNGHLVNCDTWMNIHLREVICTSKDGDRFWRMPECYIRGNTIKYLRVPDEVIDKVQEEK-(46aa)---------------------------------------------
O.sativa      MLPLSLLKTAQGHPMLVELKNGETYNGHLVNCDTWMNIHLREVICTSKDGDKFWRMPECYIRGNTIKYLRVPDEVIDKVQEET-(64aa)---------------------------------------------
S.pombe       MLPLTLLNATQGRPILVELKNGETFNGHLENCDNYMNLTLREVIRTMPDGDKFFRLPECYIRGNNIKYLRIQDEVLSQVAKQQ-(38aa)---------------------------------------------
C.neoformans  MLPLSLLTAAQGKPMLVELKNGVTFNGHLVECDTFMNVTLREVYQTSADGERFWKMKEMFIKGNIIKYFRIADNILEQAAEEQ-(64aa)---------------------------------------------
N.crassa      MLPLGILTAAQGHPMLVELKNGETLNGHLIQCDTWMNLTLREVVQTSPEGDKFVRLPEVYVKGNNIKYLRVPDDVIDIAREQQ-(47aa)---------------------------------------------
C.glabrata    MLPLYLLTNAKGQQMRIELKNGDIVEGELTNVDNWMNLTLANV-(15aa)-KVVKSKEIYVRGVYIKYITLQDDIIEKVKQQI-(80aa)---------------------------------------------
S.cerevisiae  MLPLYLLTNAKGQQMQIELKNGEIIQGILTNVDNWMNLTLSNV-(18aa)-KAVKLNEIYIRGTFIKFIKLQDNIIDKVKQQI-(94aa)---------------------------------------------
              **** :*. ::.: : :*****   :* * . *.:**: * :*        :  :  * :::*  **:: : *:::. . ::
```

```
Lsm5         1        10        20        30        40        50        60        70        80        90
             |        |         |         |         |         |         |         |         |         |
H.sapiens    MAANATTNPSQLLPLELVDKCIGSRIHIVMKSDKEIVGTLLGFDDFVNMVLEDVTEFEITPEGRRITKLDQILLNGNNITMLVPGGEGPEV---
B.taurus     MAANATTNPSQLLPLELVDKCIGSRIHIVMKSDKEIVGTLLGFDDFVNMVLEDVTEFEITPEGRRITKLDQILLNGNNITMLVPGGEGPEV---
S.scrofa     MAANATTNPSQLLPLELVDKCIGSRIHIVMKSDKEIVGTLLGFDDFVNMVLEDVTEFEITPEGRRITKLDQILLNGNNITMLVPGGEGPEV---
F.catus      MAANATTNPSQLLPLELVDKCIGSRIHIVMKSDKEIVGTLLGFDDFVNMVLEDVTEFEITPEGRRITKLDQILLNGNNITMLVPGGEGPEV---
C.familiaris MAANATTNPSQLLPLELVDKCIGSRIHIVMKSDKEIVGTLLGFDDFVNMVLEDVTEFEITPEGRRITKLDQILLNGNNITMLVPGGEGPEV---
G.gallus     MAANASTNPSQLLPLELVDKCIGSRIHIVMKSDKEIVGTLLGFDDFVNMVLEDVTEFEITPEGRRITKLDQILLNGNNITMLVPGGEGPEV---
D.rerio      MAAITATNPSQLLPLELVDKCIGSRIHIVMKNDKEIVGTLLGFDDFVNMVLEDVTEFEITPEGRRITKLDQILLNGNNITMLIPGGEGPEV---
X.laevis     MAATVPPNSSQLLPLELVDKCIGSRIHIVMKSDKEIVGTLLGFDDFVNMVLEDVTEFEITPEGRRITKLDQILLNGNNITMLVPGGEGPEV---
A.thaliana   ----MANNPSQLLPSELIDRCIGSKIWVIMKGDKELVGILKGFDVYVNMVLEDVTEYEITAEGRRVTKLDQILLNGNNIAILVPGGSPEDGE--
O.sativa     ---MSQNNPSQLLPSELIDRCIGSKIWVIMKGDKELVGTLCGFDVYVNMVLEDVTEYEYTAEGRRITKLDQILLNGNNIAILVPGGSPPDVA--
S.pombe      -------MSMTILPLELIDKCIGSNLWVIMKSEREFAGTLVGFDDYVNIVLKDVTEYDTVTGV--TEKHSEMLLNGNGMCMLIPGGKPE-----
C.neoformans -------MASTILPLELVDRCIGSPIWVLMKNEREFTGTLMGFDDYVNMVLKDVKEYEVTASGITETDLGDTLLNGNNIAMLIPGGKGPKA---
N.crassa     -------MASQLLPLELIDKCVGSRIWVVMKGDKEFAGTLVGFDDYVNMVLEDVTEFDYSGN---HTKLKKILLNGNNICMLIPGGEGPIAASA
C.glabrata   ------MSSLDVLPLEVIDKTIDQQVHIILQSNREFSGKLVGFDEFVNVILEDPIEY-(15aa)-MEHHGRMLLSGNNITMLVPGGKKL-----
S.cerevisiae ------MSLPEILPLEVIDKTINQKVLIVLQSNREFEGTLVGFDDFVNVILEDAVEW-(13aa)-MQHHGRMLLSGNNIAILVPGGKKTPTEAL
                        :** *::*: :.. : ::::.::*: * * *** :**::*:* *:         .    **.**.: :*:***.

Lsm6         1        10        20        30        40        50        60        70        80
             |        |         |         |         |         |         |         |         |
H.sapiens    MSLRKQTPSDFLKQIIGRPVVVKLNSGVDYRGVLACLDGYMNIALEQTEEYVNGQLKNKYGDAFIRGNNVLYISTQKRRM----
B.taurus     MSLRKQTPSDFLKQIIGRPVVVKLNSGVDYRGVLACLDGYMNIALEQTEEYVNGQLKNKYGDAFIRGNNVLYISTQKRRM----
S.scrofa     MSLRKQTPSDFLKQIIGRPVVVKLNSGVDYRGVLACLDGYMNIALEQTEEYVNGQLKNKYGDAFIRGNNVLYISTQKRRM----
F.catus      MSLRKQTPSDFLKQIIGRPVVVKLNSGVDYRGVLACLDGYMNIALEQTEEYVNGQLKNKYGDAFIRGNNVLYISTQKRRM----
C.familiaris MSLRKQTPSDFLKQIIGRPVVVKLNSGVDYRGVLACLDGYMNIALEQTEEYVNGQLKNKYGDAFIRGNNVLYISTQKRRM----
G.gallus     MSLRKQTPSDFLKQIIGRPVVVKLNSGVDYRGVLACLDGYMNIALEQTEEYVNGQLKNKYGDAFIRGNNVLYISTQKRRM----
D.rerio      MSLRKQTPSDFLKQIIGRPVVVKLNSGVDYRGVLACLDGYMNIAVEQTEEYVNGQLKNKYGDAFLRGNNVLYISTQKRKM----
X.laevis     MSLRKQTPSDFLKQIIGRPVVVKLNSGVDYRGVLACLDGYMNIALEQTEEYVNGQLKNKYGDAFIRGNNVLYISTQKRRM----
A.thaliana   (14aa)-PADFLKSIRGRPVVVKLNSGVDYRGTLTCLDGYMNIAMEQTEEYVNGQLKNKYGDAFIRGNNVLYISTVNMTVADGA
O.sativa     (20aa)-PSDFLKSIRGRPVVVKLNSGVDYRGILACLDGYMNIAMEQTEEYVNGQLKNKYGDAFIRGNNVLYISTSKRTLTDDA
S.pombe      ---MDSSPNEFLNKVIGKKVLIRLSSGVDYKGILSCLDGYMNLALERTEEYVNGKKTNVYGDAFIRGNNVLYVSALDD------
C.neoformans (16aa)-PSEFLRNIVGKRVKVRIGSGVDYTGLLTCLDGYMNVALEQAEEWAGEVKTAAYGDCFLRGNNVLYISALEDL-----
N.crassa     (13aa)-PSGFLSEIIGNPVTVKLNSGVVYKGELQSVDGYMNIALEKTEEFINGVKRRSYGDAFVRGNNVMYISAD--------
C.glabrata   -MAEESVSTQFLSNIIGKPVHVKLYSGMLYSGVLESIDGFMNVALSETRE-(12aa)-YASDVFLRGTQVMYISEA--------
S.cerevisiae (11aa)-TTEFLSDIIGKTVNVKLASGLLYSGRLESIDGFMNVALSSATE-(12aa)-FNNDVFLRGTQVMYISEQKI------
                       ** .: *. * ::: **: * * * .:**:**:*:. : *  .       .* *:**.:*:*:*

Lsm7         1        10        20        30        40        50        60        70        80        90        100
             |        |         |         |         |         |         |         |         |         |         |
H.sapiens    MADKEKKKKESILDLSKYIDKTIRVKFQGGREASGILKGFDPLLNLVLDGTIEYMRDPDDQYKLTEDTRQLGLVVCRGTSVVLICPQDGMEAIPNPFIQQQDA-
B.taurus     MADKEKKKKESILDLSKYIDKTIRVKFQGGREASGILKGFDPLLNLVLDGTIEYMRDPDDQYKLTEDTRQLGLVVCRGTSVVLICPQDGMEAIPNPFIQQQDA-
S.scrofa     MADKEKKKKESILDLSKYIDKTIRVKFQGGREASGILKGFDPLLNLVLDGTIEYMRDPDDQYKLTEDTRQLGLVVCRGTSVVLICPQDGMEAIPNPFIQQQDA-
F.catus      MADKEKKKKESILDLSKYIDKTIRVKFQGGREASGILKGFDPLLNLVLDGTVEYMRDPDDQYKLTEDTRQLGLVVCRGTSVVLICPQDGMEAIPNPFIQQQDA-
C.familiaris MADKEKKKKESILDLSKYIDKTIRVKFQGGREASGILKGFDPLLNLVLDGTVEYMRDPDDQYKLTEDTRQLGLVVCRGTSVVLICPQDGMEAIPNPFIQQQDA-
G.gallus     (20aa)-KKESILDLSKYIGKTIRVKFQGGREASGVLKGFDPLLNLVLDGTIEYMRDPDDQYKLTEDTRQLGLVVCRGTSVVLICPQDGMEAIPNPLIQQQDG-
D.rerio      MADKDKKKKESIFDLSKYIDKHIRVKFQGGREASGVLKGFDPLLNLVLDGTIEYMRDPDDQYKLTEDTRQLGLVVCRGTSVVLICPQDGMEAIPNPFIQQQDG-
X.laevis     MADKEKKKKESILDLSKYIDKTIRVKFQGGREASGVLKGFDPLLNLVLDGTIEYMRDPDDQYKLTEDTRQLGLVVCRGTSVVLICPQDGMEAIPNPFVQQQEG-
A.thaliana   ----MSGRKETVLDLAKFVDKGVQVKLTGGRQVTGTLKGYDQLLNLVLDEAVEFVRDHDDPLKTTDQTRRLGLIVCRGTAVMLVSPTDGTEEIANPFVTAEAV-
O.sativa     ----MSGRKETVLDLAKFVDKGVQVKLTGGRQVTGTLKGYDQLLNLVLDEAVEFEREQDDPLKLSGKTRQLGLIVCRGTAVMLVSPTDGTDEIANPFQSDGA--
S.pombe      (20aa)-RKESILDLSRYQDQRIQATFTGGRQITGILKGFDQLMNLVLDDVEEQLRNPEDG-KLTGAIRKLGLVVVRGTTLVLIAPMDGSEEIPNPFVQAE---
C.neoformans (21aa)-KRESILNLAQFVDKSIRVKFMGGREATGILKGYDQLMNLVMDDVVEEYEDG-------RPTRSLGLVVLRGPNIVLVSPTDGSSEIENPFQQ-----
N.crassa     (50aa)-KKENILDLKKYMDQRITVKFNGGREVTGTLKGYDALMNLVLDDVQEAVRDEEGN----ETTRPLGLVVARGTLLVVISPVDGSEVIANPFAQQEEEA
C.glabrata   (26aa)-KREAIVDLGKYKDSKVSVKLMGGRIVTGILKGYDQLMNLVLEETEETLKPEVETGATQQVTRQLGLTVIRGTILISLSPLDGSEVLYIQTN------
S.cerevisiae (22aa)-KREAILDLAKYKDSKIRVKLMGGKLVIGVLKGYDQLMNLVLDDTVEYMSNP-(11aa)-NARKLGLTVIRGTILVSLSSAEGSDVLYMQK-------
                    ::* :.:* :: .. : ..: **:   * ***:* *:***:: . *               * *** * **  :: :.  :* . :
```

```
Lsm8            1         10        20        30        40        50        60        70        80        90
                |         |         |         |         |         |         |         |         |         |
H.sapiens    MTSALENYINRTVAVITSDGRMIVGTLKGFDQTINLILDESHERVFSSSQGVEQVVLGLYIVRGDNVAVIGEIDEETDSALDLGNIRAEPLNSVAH---------------------------
B.taurus     MTSALENYINRTVAVITSDGRMIVGTLKGFDQTINLILDESHERVFSSSQGVEQVVLGLYIVRGDNVAVIGEIDEETDSALDLGNIRAEPLNSVAH---------------------------
S.scrofa     MTSALENYINRTVAVITSDGRMIVGTLKGFDQTINLILDESHERVFSSSQGVEQVVLGLYIVRGDNVAVIGEIDEETDSALDLGNIRAEPLNSVAH---------------------------
F.catus      MTSALENYINRTVAVITSDGRMIVGTLKGFDQTINLILDESHERVFSSSQGVEQVVLGLYIVRGDNVAVIGEIDEETDSALDLGNIRAEPLNSVAH---------------------------
C.familiaris MTSALENYINRTVAVITSDGRMIVGTLKGFDQTINLILDESHERVFSSSQGVEQVVLGLYIVRGDNVAVIGEIDEETDSALDLGNIRAEPLNSVAH---------------------------
G.gallus     MTSALENYINRTVAVITSDGRMIVGTLKGFDQTINLILDESHERVFSSSQGVEQVVLGLYIVRGDNVAVIGEIDEETDSALDLGNIRAEPLNSVVH---------------------------
D.rerio      MSTALESYIHRTVAIVTSDGRMIVGTLKGFDQAINLILDESHERVFSSSQGVEQVVLGLYIVRGDNVAVIGEIDEETDSALDLGNIRAEPLNSVVH---------------------------
X.laevis     MASALENYINRTVAVITADGRMIVGTLKGFDQTINLILDESHERVFSSSQGVEQVVLGLYIVRGDNVAVIGEIDEETDSSLDLGNIRAEPLNSVVN---------------------------
A.thaliana   (13aa)-LVDQIISVITNDGRNIVGVLKGFDQATNIILDESHERVFSTKEGVQQHVLGLYIIRGDNIGVIGELDEELDASLDFSKLRAHPLKPVVH---------------------------
O.sativa     (9aa)-SLVDQIISVITNDGRNIVGTLRGFDQATNIILDESHERVYSTREGVQQLVLGLYIIRGDNISVVGEVDEELDARLDLSNLRAHPLKPVIH---------------------------
S.pombe      --MSLADFMEQRVQVITNDGRVVLGSLKGFDHTTNLILSDSFERIISMDQDMETIPLGVYLLRGENVAMVGLVNEELDSEIEWTKIRGEAIPDVVH---------------------------
C.neoformans -MASIESYVDHTVQVILQDGRVIVGKLKGYDPRTNLILSDSVEREFSMDQGVEMIPLGLYVIKGDNVAVVAELDEEKDSTINYNDIRAEPLAELRY---------------------------
N.crassa     (7aa)-SYLNKKVCIITVDGRTLVGTLISVDMSTNVFLQRAVERVI-(11aa)-IELGTHMIRGDTVCLVGLVDEPLDESIDWTKVKGATIGTTKH---------------------------
C.glabrata   MSPLLKQYLNKDIVVVTTAGEVMHVILDGYDKYTNLVVKEG---------------DKIRLLRGSEIVVCGLLEDAKALEGLPMDS---HVYKDTKNVIKDEYLIWEAVNKKHQSTHKKRKLK
S.cerevisiae MSATLKDYLNKRVVIIKVDGECLIASLNGFDKNTNLFITNVFNRISKE------FICKAQLLRGSEIALVGLIDAENDDSLAPIDEKKVPMLKDTKNKIENEHVIWEKVYESKTK--------
                 :   :.: : ::   *. :   * . *   *:.:                      ..:* .:.. : ::
```

Supplementary Table 1 Diffraction data collection and structure refinement statistics

	U6 snRNP nt. 30-112 with 2′ PO_3	U6 snRNP nt. 30-113 with 3′ PO_3
PDB ID	5VSU	6ASO
**Data Collection**		
Wavelength (Å)	0.9792	0.9786
Resolution range (Å)*	179.8-3.10 (3.29-3.10)	90.59-2.80 (2.91-2.80)
Space group	$P2_12_12_1$	$P2_1$
Unit cell dimensions (Å)	70.2, 114.7, 179.8	94.2, 77.4, 118.6
		β = 105.9°
Total reflections*	1,389,951 (206,204)	2,475,089 (267,471)
Unique reflections*	27,164 (4,321)	40,745 (4,574)
Multiplicity*	51.2 (47.7)	60.7 (58.5)
Completeness (%)*	100 (100)	100 (100)
Mean I/σ(I)*	13.0 (1.2)	8.7 (0.5)
Wilson B-factor ($Å^2$)	129.7	79.1
anisotropic ΔB ($Å^2$)	17.4	87.2
R_{merge}*	0.25 (4.03)	0.45 (16.6)
R_{pim}*	0.035 (0.59)	0.058 (2.18)
$CC_{1/2}$*	1.00 (0.445)	0.997 (0.418)
**Refinement**		
Resolution	96.7-3.10	55.6-2.71
R_{work}/R_{free}*	0.234/0.298	0.195/0.247
Total number of atoms	9,240	8,863
macromolecules	9,240	8,724
ligands	0	5
water	0	134
RMS(bonds)	0.011	0.011
RMS(angles)	1.667	1.656
Ramachandran favored	93.1	94.8
Ramachandran outliers	1.81	1.02
Average B factor ($Å^2$)	133.4	79.5
macromolecules	133.4	79.5
ligands/ions	NA	74.5
solvent	NA	45.6

*Values shown in parentheses are for the highest resolution shell. Isotropic data statistics are only shown to 2.8 Å for the anisotropic 3′ PO_3 dataset, where ellipsoidal truncation was used to generate the final dataset for structure refinement with resolution limits of 2.71 x 3.50 x 3.59 Å. Ellipsoidal truncation was performed on the StarAniso web server with the default I/σ(I) cutoff of 1.2.

Supplementary Table 2 Growth rate of wild-type and *prp24* strains of *S. cerevisiae* in liquid media

	doubling time in YEPD (minutes)	
strain	30 °C	37 °C
PRP24	94 ± 3	88 ± 4
prp24-8Asub	112 ± 5	150 ± 9
prp24-ΔSNFFL	106 ± 1	114 ± 1
prp24-8Asub-ΔSNFFL	132 ± 0	188 ± 6

Supplementary Table 3 Binding affinity of wild-type and mutant Lsm2-8 for the 3′ region of *S. cerevisiae* U6 RNA (nucleotides 104-113) with either a 2′,3′-*cis* diol or a 3′ phosphate.

	diol	PO_3	K_d ratio diol/PO_3	K_d ratio PO_3/diol
WT	37.4 ± 4.5	6.13 ± 0.87	6.11	0.16
Lsm2-K20A	522 ± 48	841 ± 89	0.62	1.61
Lsm2-K20E	1,870 ± 370	2,320 ± 450	0.81	1.24
Lsm3-R21A	40.8 ± 3.3	123 ± 12	0.33	3.01
Lsm3-R21E	195 ± 27	1,150 ± 190	0.17	5.89
Lsm8-6Asub	132 ± 16	301 ± 33	0.44	2.29
Lsm8-delC	277 ± 32	502 ± 47	0.55	1.81

Affinities are reported as K_d values with units of nM. Binding data were obtained via fluorescence polarization with 5′ FAM labeled oligonucleotides (Fig. 5) and are from two technical replicates.

Supplementary References

1. Didychuk, A.L., Montemayor, E.J., Brow, D.A. & Butcher, S.E. Structural requirements for protein-catalyzed annealing of U4 and U6 RNAs during di-snRNP assembly. *Nucleic Acids Res* **44**, 1398-410 (2016).
2. Rader, S.D. & Guthrie, C. A conserved Lsm-interaction motif in Prp24 required for efficient U4/U6 di-snRNP formation. *RNA* **8**, 1378-92 (2002).
3. Achsel, T. et al. A doughnut-shaped heteromer of human Sm-like proteins binds to the 3'-end of U6 snRNA, thereby facilitating U4/U6 duplex formation in vitro. *EMBO J* **18**, 5789-802 (1999).
4. Montemayor, E.J. et al. Core structure of the U6 small nuclear ribonucleoprotein at 1.7-A resolution. *Nat Struct Mol Biol* **21**, 544-51 (2014).
5. Zhou, L. et al. Crystal structures of the Lsm complex bound to the 3' end sequence of U6 small nuclear RNA. *Nature* **506**, 116-20 (2014).
6. Li, J., Leung, A.K., Kondo, Y., Oubridge, C. & Nagai, K. Re-refinement of the spliceosomal U4 snRNP core-domain structure. *Acta Crystallogr D Struct Biol* **72**, 131-46 (2016).
7. Li, X. et al. CryoEM structure of Saccharomyces cerevisiae U1 snRNP offers insight into alternative splicing. *Nat Commun* **8**, 1035 (2017).
8. Wilusz, C.J. & Wilusz, J. Lsm proteins and Hfq: Life at the 3' end. *RNA Biol* **10**, 592-601 (2013).
9. Dimastrogiovanni, D. et al. Recognition of the small regulatory RNA RydC by the bacterial Hfq protein. *Elife* **3**(2014).
10. Someya, T. et al. Crystal structure of Hfq from Bacillus subtilis in complex with SELEX-derived RNA aptamer: insight into RNA-binding properties of bacterial Hfq. *Nucleic Acids Res* **40**, 1856-67 (2012).
11. Updegrove, T.B., Zhang, A. & Storz, G. Hfq: the flexible RNA matchmaker. *Curr Opin Microbiol* **30**, 133-138 (2016).
12. Sharif, H. & Conti, E. Architecture of the Lsm1-7-Pat1 complex: a conserved assembly in eukaryotic mRNA turnover. *Cell Rep* **5**, 283-91 (2013).
13. Spiller, M.P., Reijns, M.A. & Beggs, J.D. Requirements for nuclear localization of the Lsm2-8p complex and competition between nuclear and cytoplasmic Lsm complexes. *J Cell Sci* **120**, 4310-20 (2007).
14. Ashkenazy, H. et al. ConSurf 2016: an improved methodology to estimate and visualize evolutionary conservation in macromolecules. *Nucleic Acids Res* **44**, W344-50 (2016).
