# Supplementary material for: Architecture of the U6 snRNP reveals specific recognition of 3′-end processed U6 snRNA
Source: Nat Commun. 2018 May 1;9:1749. doi: 10.1038/s41467-018-04145-4 (PMC5931518; doi:10.1038/s41467-018-04145-4)
Supplement: Supplementary file 3 — Description of Additional Supplementary Files [file 41467_2018_4145_MOESM3_ESM.pdf]

Description of Additional Supplementary Files:

Supplementary Movie 1: **Structure of the U6 snRNP.** This video shows a 360 degree rotation of the U6 snRNP (PDB coordinate file: 5VSU). Colors are as in Figure 1.

Supplementary Dataset 1: Pymol session file of the overlayed U6 snRNP structures (PDB coordinate files: 6ASO and 5VSU). Colors are as in Figure 1.
